# Community Pharmacy Turnover and Context of Openings and Closings by Ownership Type

**DOI:** 10.1001/jamahealthforum.2025.1988

**Published:** 2025-08-01

**Authors:** T. Joseph Mattingly, Maitreyi Sahu, Kelly E. Anderson

**Affiliations:** 1 Department of Pharmacotherapy, University of Utah College of Pharmacy, Salt Lake City; 2 Department of Health Metrics Sciences, University of Washington, Seattle; 3 Department of Clinical Pharmacy, University of Colorado Skaggs School of Pharmaceutical Sciences, Aurora

## Abstract

**Importance:**

While community pharmacies provide many valuable prescription and professional services, they are also retail businesses. Evaluating pharmacy closures through the lens of a retail business framework may provide more context on what is happening in this industry and identify potential solutions to address closures and/or shortages.

**Objective:**

To evaluate community pharmacy turnover (openings and closings) in the US over time and by ownership type.

**Design and Setting:**

This cross-sectional analysis of all community pharmacy openings and closings in the US from 2010 to 2023 used pharmacy-level data from the US National Council for Prescription Drug Programs database. National-, state-, and county-level turnover was assessed using economic indicators of business dynamics, such as total population, population growth, household income, total firm changes for all industries, and net job creation.

**Exposures:**

Pharmacy class type defined as either a chain pharmacy (4 or more pharmacies under common ownership) or an independent or franchise pharmacy using the US National Council for Prescription Drug Programs classifications.

**Main Outcomes and Measures:**

Pharmacy turnover rate from 2010 to 2023, calculated as the sum of pharmacy openings and closings over the full study period divided by the total pharmacies in the market at the beginning of the period (2010).

**Results:**

The analyses found that US pharmacy market turnover rate for this 14-year period was 86.8% (52 974 total openings and closures of 61 054 total pharmacies in 2010) or 6.2% annually. When comparing across pharmacy types, independent pharmacy turnover was substantially higher (152.7%) than chain pharmacy turnover (49.9%) across the entire US. Counties with high turnover were associated with net increases in pharmacies from more independent pharmacy openings over the period. Turnover rates for all businesses were higher in counties with both low and high pharmacy turnover, even when adjusting for population size.

**Conclusions and Relevance:**

This cross-sectional study found that the community pharmacy market in the US has an annual turnover rate of approximately 6.2%, with independent pharmacies opening and closing more frequently than chain pharmacies.

## Key Points

**Question:**

Does community pharmacy turnover—both openings and closures—vary by pharmacy ownership type and what are the observable market patterns over time?

**Findings:**

This cross-sectional study including more than 60 000 community pharmacies found that during a 14-year time period, the total turnover rate was 86.8%, or approximately 6.2% annually, with independent pharmacies opening and closing more frequently than chain pharmacies. High turnover was associated with more independent pharmacy openings, and chain pharmacies experienced consistent net losses after 2015, whereas net changes for independent and franchise pharmacies remained flat.

**Meaning:**

These findings indicate that independent pharmacies represent a larger proportion of both openings and closings with a relatively flat net change, a turnover trend that may reflect typical retail industry dynamics; however, more research is needed to understand the downstream implications for patients and communities.

## Teaser

This cross-sectional study evaluates pharmacy openings and closures by pharmacy ownership type during a 14-year period in the US.

## Introduction

Community pharmacy in the US has undergone a substantial transformation in the past century, evolving beyond the soda fountain era of the 1920s—when pharmacies often served as social and community hubs—to the current pharmaceutical care era in which pharmacists and pharmacy technicians dispense medications, administer vaccines, screen for chronic diseases, counsel patients, and in some cases, even prescribe medications.^1,2^ In recognition of this important role, brick and mortar pharmacies are considered a critical part of our health care infrastructure. However, community pharmacies also operate as retail businesses, balancing health care delivery with consumer-oriented sales. More than three-fifths of community pharmacies are part of large chains (eg, CVS, Walgreens, Walmart),^3^ and many generate their highest profit margins not from prescription drugs but rather from front-end sales, such as over-the-counter medicines, cosmetics, household supplies, and food products. Accordingly, community pharmacies face both health care−specific and broader retail market pressures.

Recently, concerns have been raised by researchers,^4^ lay press,^5^ and policymakers^6^ that community pharmacy closures are leading to a lack of access or driving potential health disparities.^7,8,9^ Two studies have found associations between pharmacy closures and reductions in patient utilization of prescription drugs in the immediate period after a closure, eg, for cardiovascular medications^10^ and anticonvulsants.^11^ However, the impact of pharmacy closures on longer-term access to and affordability of medicines, health system costs, patient health, and health disparities remain poorly understood. In addition, while much attention has focused on insurer reimbursement practices creating financial pressure for independent pharmacies, this period has also coincided with shifting market dynamics including ongoing openings and closures for both independent and chain pharmacies,^9^ substantial consolidation across the health care industry, shifts in consumer preferences toward e-commerce, and the establishment of direct-to-consumer online pharmacies.

Openings, closings, and turnover are frequently described in business, marketing, and operational research as *churn* and can refer to either customer churn or business churn. The most common churn analysis^12^ focuses on consumer behaviors and the phenomenon of a consumer switching from a company to another. Churn has also been applied to business-level analyses to evaluate business openings and closures in different markets.^13,14^ The literature on retail store churn and retail business disruption can provide some insight for the community pharmacy industry.^14^ To remain afloat, retailers must continuously evaluate store performance, monitor neighborhood development and population changes, react to changing consumer demands, and consider impacts from competition and increasing retail density.^14^ For example, mail-order pharmacy services have been available since the 1940s and vertically integrated payers have used these channels to steer prescription volume away from traditional brick-and- mortar retail pharmacies.^15^ While business-level churn can represent a healthy marketplace with entrepreneurs starting new businesses or existing businesses expanding locations, churn can also represent a declining market or area where businesses are struggling to maintain a profitable operation or attract customers. Given the prior work on medication adherence after a pharmacy closure,^10,11^ high churn rates for pharmacies may be of concern to health systems and policymakers.

This study sought to evaluate pharmacy closures and openings from 2010 to 2023, using the concept of business churn rate. We aimed to describe patterns for both openings and closures, disaggregated by type of pharmacy ownership (independent and franchise or chain). We also assessed pharmacy turnover at county- and state-levels to explore other economic factors. By detailing the circumstances of openings and closures in addition to pharmacy characteristics associated with overall churn rate, we aimed to build context around the discussion of pharmacy closures.

## Methods

This study was reviewed by the institutional review board of the University of Utah. Informed consent was waived because the study did not include any human participants. We reported our methods and results in accordance to best practices for reporting from the Strengthening the Reporting of Observational Studies in Epidemiology (STROBE) reporting guideline.

### Study Design, Data Sources, and Sample

We conducted a cross-sectional analysis of all community pharmacy openings and closings from 2010 to 2023 using data from the US National Council for Prescription Drug Programs’ (NCPDP) dataQ product. The NCPDP is a nonprofit, multistakeholder forum that develops standards and business solutions for electronic transactions for pharmacies including real-time claims adjudication, eligibility and benefit verification, and sharing of medication history. NCPDP maintains data on more than 75 000 pharmacy and nonpharmacy dispensing sites, with an NCPDP Provider Identification Number provided to every licensed pharmacy in the US and its territories.^16^ Only community and retail pharmacy classifications were included in this analysis (long-term care, mail-order, specialty, clinic, and any others were excluded) and only independent, chain, and franchise pharmacy dispenser types were included (government and alternate dispensing sites were excluded).

### Churn Measures

The primary outcome variable was pharmacy churn rate from 2010 to 2023, defined as the sum of pharmacy openings and closings during this time period (14 years) divided by the total pharmacies in the market at the beginning of the period (2010).^14^ Using the NCPDP record, pharmacy open date and close date were used to identify pharmacy openings and closures during the time period. Because new NCPDP numbers could represent a pharmacy acquisition or a pharmacy using multiple NCPDP numbers for different billing practices, we identified the specific physical location where the NCPDP number was assigned. Using street address, city, state, and zip code, we identified unique addresses with just 1 NCPDP record and addresses where multiple records were assigned over the period.

### Exposure

Our primary exposure was the pharmacy class type defined as either a chain or an independent or franchise pharmacy using NCPDP classification. A chain pharmacy is defined by NCPDP as a group of 4 or more pharmacies under common ownership.^16^ We first evaluated nationwide churn by pharmacy class type per year and then further evaluated these variables by state and by county.

### Covariates

In addition to pharmacy ownership type, we explored independent variables such as rural status (using modified Rural-Urban Continuum Codes defined by the US Department of Agriculture),^17^ county population size, county population change, changes in the total number of firms in each county, and county net job creation from the US Census Bureau and the Business Dynamic Statistics database.^18^ The latter provides detailed information on firm openings, closures, expansions, contractions, and net job creation, data that were used as measures of broader business dynamics to contextualize pharmacy churn given that pharmacies operate within local economic ecosystems. Understanding broader business entry and exit trends can offer important context for evaluating pharmacy-level market stability and volatility at the state and county levels. We operationalized these variables as continuous variables.

### Statistical Analysis

First, we described the full sample and conducted χ^2^ tests to test the associations between the primary exposure (pharmacy ownership group) and whether the pharmacy opened, closed, or opened and closed during the observation period. Next, we created visualizations of net pharmacy openings and closures by year to describe trends over time. Additionally, we calculated pharmacy churn rates at the state and county levels. For the county-level analysis, we evaluated counties by categorizing each county as either high or low pharmacy churn counties based on the median churn over the full study period. For each continuous variable, the Wilcoxon test was used because the Shapiro-Wilk normality test indicated that at least 1 of the groups (low churn or high churn) deviated significantly from normality (*P* ≤ .05). All analyses were performed using R, version 12.1 (R Foundation for Statistical Computing).^19^

## Results

For 2010, we identified 61 054 retail pharmacies (39 158 chain; 21 896 independent/franchise) and at the end of 2023, there were 60 354 retail pharmacies (36 622 chain; 23 732 independent/franchise). For 2010 to 2023, we identified 66 032 NCPDP records of retail chain or independent/franchise pharmacies that were designated as either opened or closed. Of these 66 032 NCPDP records, 34 636 (52.5%) were single opening or closure records at a unique address. The other 31 396 records were multiple records at 13 795 unique addresses. Using this multiple record data, we were able to identify 43 625 unique pharmacy locations where there had been a true opening or true closure, after excluding 4806 pharmacy locations for which the NCPDP closing record was followed by an NCPDP opening record within 30 days with no subsequent closing (eFigure in Supplement 1). These 4806 pharmacies represent acquisitions in which pharmacy operations continued; therefore, they do not represent actual business churn.

**Figure 1.**
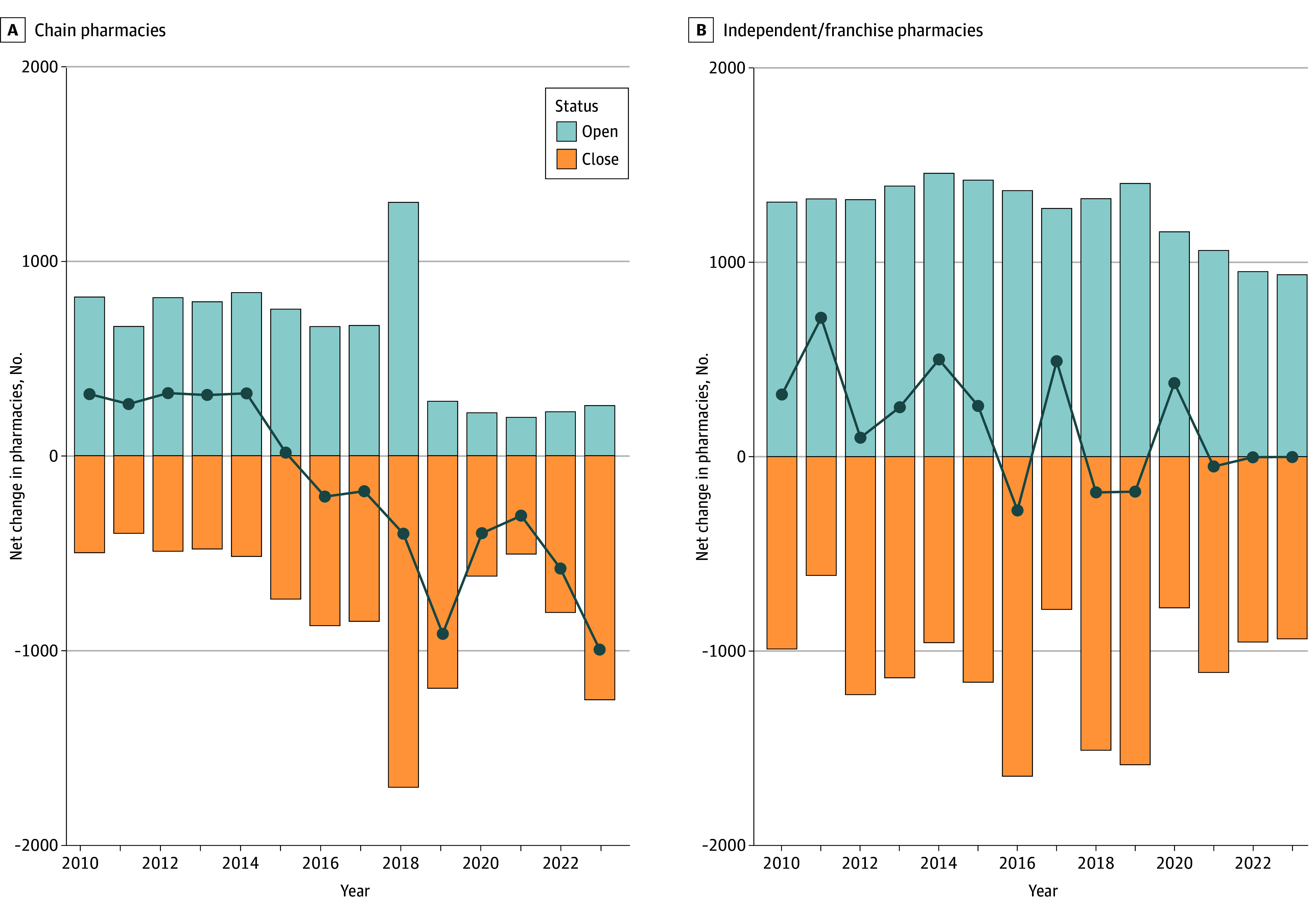
Openings and Closures of Chain Pharmacies and Independent/Franchise Pharmacies From 2010 to 2023 National retail pharmacy opening and closures at specific physical locations from 2010 to 2023 show that chain pharmacy types (defined as 4 or more pharmacies under common ownership) experienced net gains per year from 2010 to 2015 followed by net declines from 2016 to 2023. Meanwhile, independent or franchise owners experienced a relatively flat net change over the entire period with a mix of years with net gains or net losses. Circles indicate net gain or loss each year.

For the full sample of unique pharmacy locations, we identified 17 493 (40.1%) pharmacy closures, 16 783 (38.5%) pharmacy openings, and 9349 (21.4%) pharmacies that opened and truly closed during the study period (Table 1). From 2010 to 2023, total pharmacy churn (sum of all openings and closures) was 52 974 because openings with subsequent closures are counted twice. This represents a total churn rate of 86.8% during the 14-year period or 6.2% per year, considering there were 61 054 total community chain and independent/franchise pharmacies operating in 2010. When stratifying by the pharmacy ownership type, independent/franchise total churn and churn rates were substantially higher—33 438 openings and closings over the period representing a 152.7% churn rate (10.9% per year)—compared to chain pharmacies—19 536 openings and closings, representing a 49.9% churn rate (3.6% per year) (Figure 1). The primary driver for the churn difference by ownership type was the statistically significant number of openings and openings with a subsequent closure for independent/franchise pharmacies.

**Table 1.** Community Pharmacy Opening and Closure Records With Unique Address, 2010 to 2023

Characteristic	Pharmacy churn type			*P* value
	Closure (n = 17 493)^a^	Opening (n = 16 783)^b^	Openings and closures (n = 9349)^c^	
Pharmacy ownership group				
Chain^d^	8395 (48.0)	5809 (34.6)	2666 (28.5)	<.001
Independent pharmacy or franchise	9098 (52.0)	10 974 (65.4)	6683 (71.5)	
County-level rural-urban continuum code				
Large metro (>1 million population)	9463 (54.1)	10 139 (60.4)	6060 (64.8)	<.001
Medium metro (250 000-1 million population)	3.301 (18.9)	3100 (18.5)	1521 (16.3)	
Small metro (<250 000 population)	1595 (9.1)	1407 (8.4)	633 (6.8)	
Midsize nonmetro (20 000 or more)	1115 (6.4)	731 (4.4)	391 (4.2)	
Small nonmetro and rural (<20 000 population)	2019 (11.5)	1406 (8.4)	744 (8.0)	
Other county-level characteristic, mean (SD)				
Population in 2022	1 165 452 (1 904 023)	1 279 696 (1 877 678)	1 429 043 (1 865 367)	<.001
Median household income, $	69 686 (19 419)	71 022 (19 065)	69 725 (18 366)	<.001
Net job creation 2010-2022	83 996 (125 937)	95 149 (125 321)	114 886 (139 650)	<.001
Unemployment rate	9.95 (2.66)	9.86 (2.67)	9.92 (2.48)	.004

^a^ Record of closing at specific address with no subsequent opening at that address.

^b^ Record of opening at specific address.

^c^ Record of opening and closing occurring at the same address with the most recent record being a closure.

^d^ Defined as 4 or more pharmacies under common ownership.

**Figure 2.**
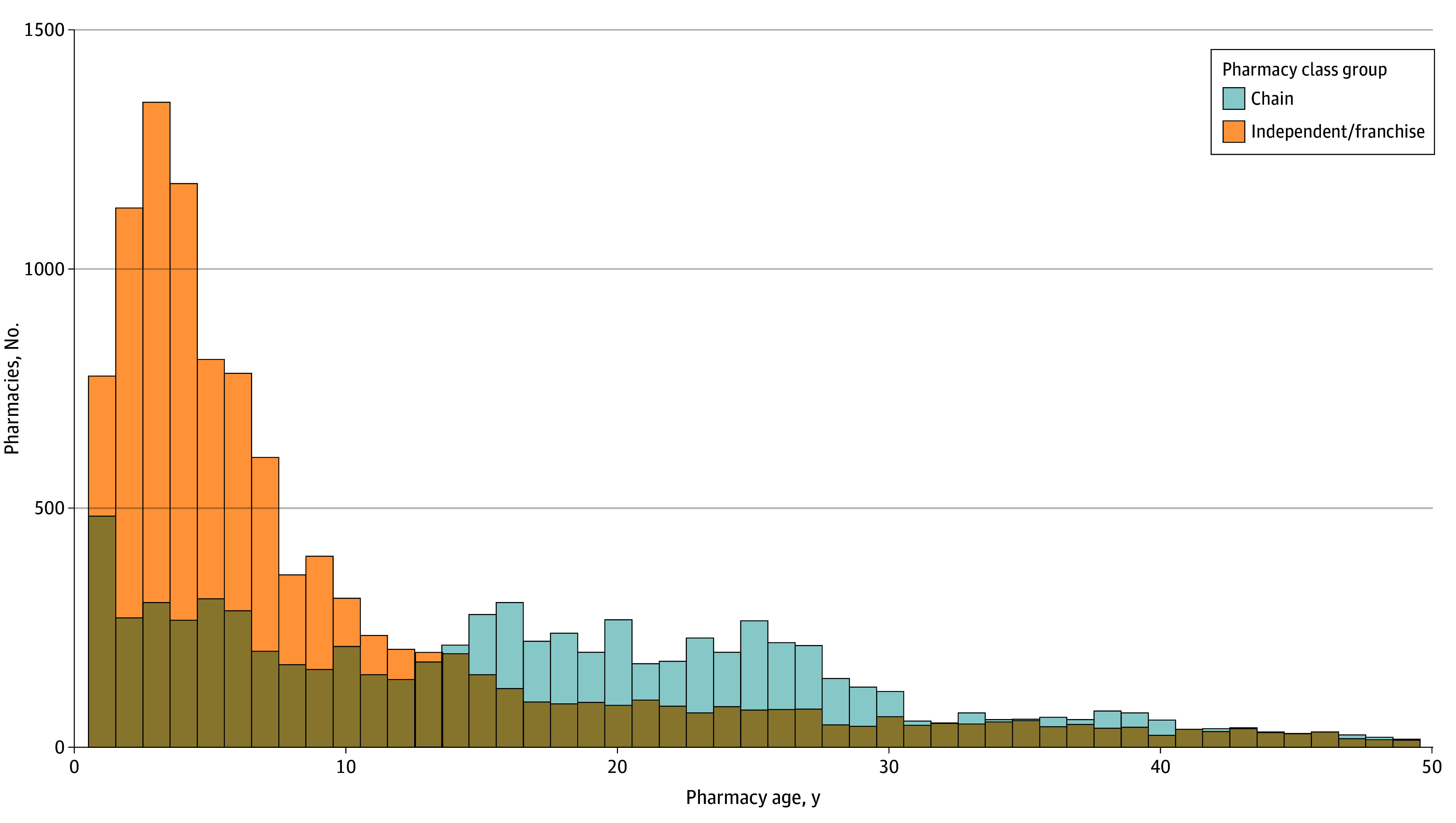
Pharmacy Age at Time of Closure for Chain and Independent or Franchise Pharmacies The frequency distribution of pharmacy age at the time of pharmacy closure reveals different patterns for independent/franchise pharmacies and chain pharmacies. Independent/franchise age data shows a substantial right tail distribution with a large frequency of closures occurring in the first 5 years of ownership with a long tail demonstrating a small number of older (>40 years) pharmacies. Chain pharmacies have a smaller spike in the first year followed by a relatively consistent frequency for 1 to 27 years of age before the distribution of closures drops.

Additionally, openings and openings with subsequent closure were more likely to occur in larger counties with more net job creation for all businesses during the same period. When visualizing the openings and closures by year (Figure 1), churn rates were considerably higher for independent/franchise pharmacies in all years except for 2018. Additionally, chains maintained consistent net openings from 2010 to 2015 followed by net closings from 2016 to 2023. Given the frequency of openings with subsequent closure rates observed for independent/franchise pharmacies, we visualized the pharmacy age (in years) for all pharmacy closure records in our sample to compare the distribution of age at time of closure by pharmacy ownership class group (Figure 2).

For our county-level analysis, we compared the bottom 50% of counties to the top 50% of counties based on observed churn over the period (Table 2). Compared to low-churn counties, high-churn counties experienced more net pharmacy openings (0.5 vs −1.0), with net pharmacy gains in these counties occurring for independent/franchise pharmacies with net chain pharmacy losses. We did not observe a statistically significant difference in county population size in 2010 or 2022 between the low- and high-churn counties or a difference in net job creation per 10 000 people. Total firm churn rates for all businesses were higher in both low and high pharmacy churn counties, even when adjusting for population size.

**Table 2.** County-Level Pharmacy Churn Analysis Adjusted Based on Population Size

Variable	Counties, mean (SD)^a^		*P* value^b^
	Low churn (n = 1497)	High churn (n = 1498)	
Pharmacies in 2010			
Total pharmacies	16.0 (36.4)	24.8 (79.0)	<.001
Chain^c^	11.3 (29.4)	14.9 (44.9)	.42
Independent/franchise	4.7 (8.0)	9.9 (38.4)	<.001
Pharmacies in 2023			
Total pharmacies	15.0 (34.9)	25.3 (82.2)	<.001
Chain	10.5 (27.3)	14.0 (41.8)	.98
Independent/franchise	4.5 (8.6)	11.3 (46.7)	<.001
Net pharmacy change from 2010-2023	−1.0 (3.9)	0.5 (12.1)	<.001
Pharmacy churn from 2010-2023	8.8 (24.3)	26.1 (95.3)	<.001
Total pharmacy churn rate	0.37 (0.3)	1.1 (0.8)	<.001
Net job creation from 2010-2022	4926 (23 999)	7855 (34 619)	.03
Total firm change from 2010-2022	131 (738)	259 (1615)	.05
Total churn, all firms	4571 (13 298)	6893 (27 463)	.70
Total churn rate, all firms	2.38 (0.5)	2.42 (0.5)	.09
Median household income, $	60 728 (15 192)	57 204 (15 216)	<.001
Adjustments for population			
County population, 2010	91 512 (222 471)	114 298 (393 502)	.10
County population, 2022	97 118 (245 076)	124 641 (412 844)	.07
Variable per 10 000 population			
Total pharmacies in 2023	1.96 (0.9)	2.45 (1.1)	<.001
Pharmacy churn	0.66 (0.5)	2.78 (1.7)	<.001
Pharmacy churn rate	0.10 (0.2)	0.76 (1.5)	<.001
Total firm change	0.13 (25.8)	−1.14 (29.2)	.006
Total churn, all firms	446 (197)	474 (216)	<.001
Churn rate, all firms	1.48 (2.0)	1.47 (1.8)	.04
Net job creation	172 (524)	147 (615)	.09

^a^ Low-churn and high-churn counties were defined based on the median county-level pharmacy churn rates observed from 2010-2023.

^b^ Wilcoxon test was used for all variables because the Shapiro-Wilk normality test indicated that at least 1 of the groups (low churn or high churn) deviated significantly from normality (*P* ≤ .05). As a nonparametric test, means (SDs) may appear similar but the distributions are skewed differently.

^c^ Defined as 4 or more pharmacies under common ownership.

**Figure 3.**
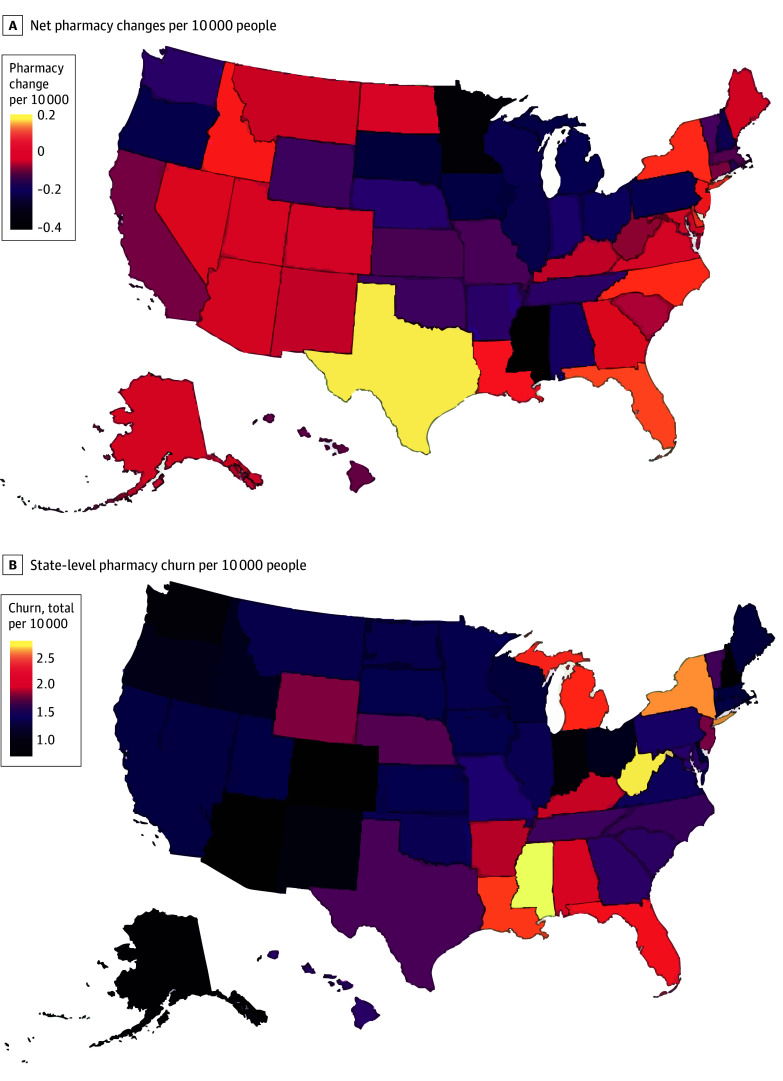
Net Pharmacy Changes and Churn per 10 000 Population A, Differences in pharmacy growth (positive net change) vs pharmacy decline (negative net change) at the state level adjusted for population. B, US states with greater churn (larger sum of openings and closures) vs states with lower churn (smaller sum of openings and closures) adjusted for population.

At the state level, positive net pharmacy growth per 10 000 people occurred in 19 states and the District of Columbia while net pharmacy declines occurred in the remaining 31 states. On a per capita basis, the District of Columbia, Texas, Florida, Delaware, and New York were the 5 biggest areas of net pharmacy growth while Mississippi, Minnesota, South Dakota, Iowa, and Michigan observed the most net pharmacy losses per capita (Figure 3A). In terms of churn, Mississippi, West Virginia, New York, Louisiana, and Michigan experienced the most total churn per 10 000 people while Colorado, Arizona, Alaska, New Hampshire, and Indiana experienced the least amount of churn per capita (Figure 3B).

## Discussion

Pharmacy closures have garnered substantial attention in recent years. Using a retail lens, we applied business churn rate methods to better understand pharmacy closures and openings across US.^14^ Our results support research by Guadamuz et al^9^ who demonstrated that since 2018 more US pharmacies have closed than have opened; however, we conclude that this is driven by chain pharmacy changes and potentially confounded by other business dynamics. Building on these results, we found that during 2010 to 2023, the total retail pharmacy churn rate was 86.8% (6.2% annually), which is in line with prior research of other industries, eg, the 7.0% of grocery retailers that either open or close annually in the US.^20^ In particular, we find the highest churn rate among independent pharmacies (152.7% [10.9% annually]), which have had more net openings than closings since 2010, and lower churn rate among chain pharmacies (49.9% [3.6% annually]), which have had more net closures than openings during this time. This finding is consistent with independent vs chain grocery store findings, where churn is more frequent in independent grocery stores as well.^20^ Accordingly, we found that prior conclusions, ie, “independent pharmacies are at greater risk for closure”^4^ compared with chain pharmacies in the context of openings appear to incompletely describe overall access to independent pharmacies over time.^9^

Our findings that independent pharmacies open as frequently as they close adds an additional layer of complexity that merits increased attention. First, this phenomenon reflects the relatively lower barrier to market entry for an independent business and potential business strategies for independent pharmacy owners. For example, given the known practices among large chain pharmacies to buy independent pharmacies, an independent pharmacy owner may aim to sell their pharmacy to a chain, and then those same pharmacy owners may open a new independent pharmacy.^21^ Based on the distribution of pharmacy age at time of closure, many independent/franchise pharmacy closures frequently occur in the first 5 years of business (Figure 2), but with a long right tail skewness capturing the pharmacy closures for long-standing businesses that are often landmarks in a small community or neighborhood. When these well-established businesses that have treated multiple generations of patients in the same town ultimately close, it may be more newsworthy and of interest to the general population than the news of 100 pharmacies closing that were simply failed attempts by a new entrepreneur.

Beyond contextualizing both openings and closures, our analysis demonstrates that focusing solely on a pharmacy opening or closing record using national- or state-level data sources, such as the NCPDP or state boards of pharmacy, may miss some of the nuance around the business dynamics happening in a community. When evaluating the NCPDP openings and closings at unique pharmacy locations, we found a substantial number of pharmacy acquisitions where a new pharmacy opening occurred at the same exact address as the closure within a short period of time. For an independent/franchise pharmacy owner, being acquired by another business is a common exit strategy that can have a financially lucrative outcome. The National Community Pharmacists Association , originally founded in 1898 as the National Association of Retail Druggists, provides “pharmacy ownership transition” tools for students and pharmacy owners to help evaluate the value of an existing pharmacy business and help determine various strategies that include both file acquisition and continuing operations.^22^

Closing an existing business and reopening under a new name is distinct from a relatively common phenomenon known as *file acquisition*, which specifically refers to patient records and prescriptions at an existing pharmacy being purchased and moved to a new, often nearby, location or in the same location under the new ownership’s brand identity.^22^ After a file acquisition, patients are not obligated to use the new pharmacy so the new owners are initially motivated to provide exceptional service to prevent patients from transferring their business elsewhere (eg, customer churn). So, it is in both the pharmacy buyer’s and seller’s best interest to ensure a smooth transition to increase the value of the files being purchased.

Given the recent announcement of chain location closures by Walgreens and CVS, we may anticipate new openings by independent or other smaller chains in areas where the largest retailers close—closure creates a new market opportunity. The news of a private equity deal to purchase Walgreens^23^ further supports the hypothesis of new market opportunities if the new private entity follows past trends in private equity deals in health care.^24^ The private entity may seek to sell off many of its assets including existing pharmacy buildings, real estate, and patient records and prescription files, as previously discussed. In other words, an entrepreneur with substantial knowledge of the local market may be able to acquire the files from an existing unprofitable Walgreens (and motivated seller) at a discounted price and then work to rebrand that location and try to make it a profitable business.

We may also anticipate retail changes in these areas given that nearby pharmacies may attempt to capture the prescription volume from these closures for patients willing to travel to their locations or if they can provide home delivery. In recent years, e-commerce has put additional pressures on all retailers culminating in the “retail apocalypse” of 2018 during which thousands of physical retail locations for companies such as Sears, Kmart, and Toys“R”Us were closed when consumer preferences shifted to online ordering and delivery.^14^ Despite these retail store challenges from e-commerce, Barnes & Noble was on track to open 58 new locations in 2024—marking the largest number of openings for the bookseller since 2009.^25^ With a thoughtful redesign focused on consumer preferences, shopper experience, and customer loyalty, physical retail pharmacy locations could evolve similarly to other retail businesses.

Ultimately, we need to understand the net costs and benefits of a retail-level of churn for pharmacies. Policies that seek to prevent pharmacy closure to avoid pharmacy deserts, medication adherence challenges, and other negative consequences associated with closures may be desirable but could come with potential unintended consequences. Policies that protect existing pharmacies from closure may prevent new businesses with more innovative pharmacy services offerings from opening. Based on the data, a way to prevent pharmacy closure would be more stringent requirements for new pharmacy licenses. While this approach may reduce the number of independent pharmacies that fail shortly after opening, it may also limit openings to only those with access to more capital (eg, hospitals, universities, major corporations, private equity).

### Limitations

Similar to other analyses using NCPDP pharmacy-level data, this analysis has several limitations including no information on pharmacy prescription volume, pharmacy revenue, pharmacy benefit manager contracts, and patient characteristics for each pharmacy. We also did not explore any downstream impact on patient adherence or health. An important limitation relevant to this analysis is that the NCPDP data may have a lag in reflecting pharmacy closures, and more current data for openings because pharmacies need to be listed with NCPDP to receive reimbursements. This disparity would mean underestimation of closures; however, our numbers are generally consistent with other studies of pharmacy closures. Additionally, this lag along with the trending decline since 2018 may suggest 2024 data could reflect more closures than openings for all pharmacy types. We also used county-level demographic and economic data to draw conclusions on these populations but also acknowledge the substantial heterogeneity within a single county based on neighborhood or local area dynamics.

## Conclusions

This cross-sectional study shows that the community pharmacy market in the US had an annual churn rate of approximately 6.2% from 2010 to 2023, with independent pharmacies opening and closing more frequently than chain pharmacies. Counties with higher rates of churn actually had net increases in total pharmacies over the period, whereas low-churn counties experienced net decreases. Additionally, total firm churn rates for all businesses were higher in both the pharmacy low- and high-churn counties, even when adjusting for population size. Researchers, health system leaders, and policymakers should be cognizant of the disruption in health care that patients may face after a pharmacy closure and evaluate all of the costs and benefits of pharmacy churn on pharmaceutical care delivery.

## Notes

Supplement 1. eFigure. STROBE flow diagram.

Supplement 2. Data Sharing Statement
