# Supplementary material for: Community Pharmacy Turnover and Context of Openings and Closings by Ownership Type
Source: JAMA Health Forum. 2025 Aug 1;6(8):e251988. doi: 10.1001/jamahealthforum.2025.1988 (PMC12317352; doi:10.1001/jamahealthforum.2025.1988)
Supplement: Supplement 1. — eFigure. STROBE flow diagram. [file jamahealthforum-e251988-s001.pdf]

Supplemental Online Content

Mattingly TJ, Sahu M, Anderson KE. Community pharmacy turnover and context of openings and closings by ownership type. *JAMA Health Forum.* Published online August 1, 2025. doi:10.1001/jamahealthforum.2025.1988

eFigure. STROBE flow diagram

This supplemental material has been provided by the authors to give readers additional information about their work.

© 2025 Mattingly TJ et al. *JAMA Health Forum*.

eFigure. STROBE flow diagram.

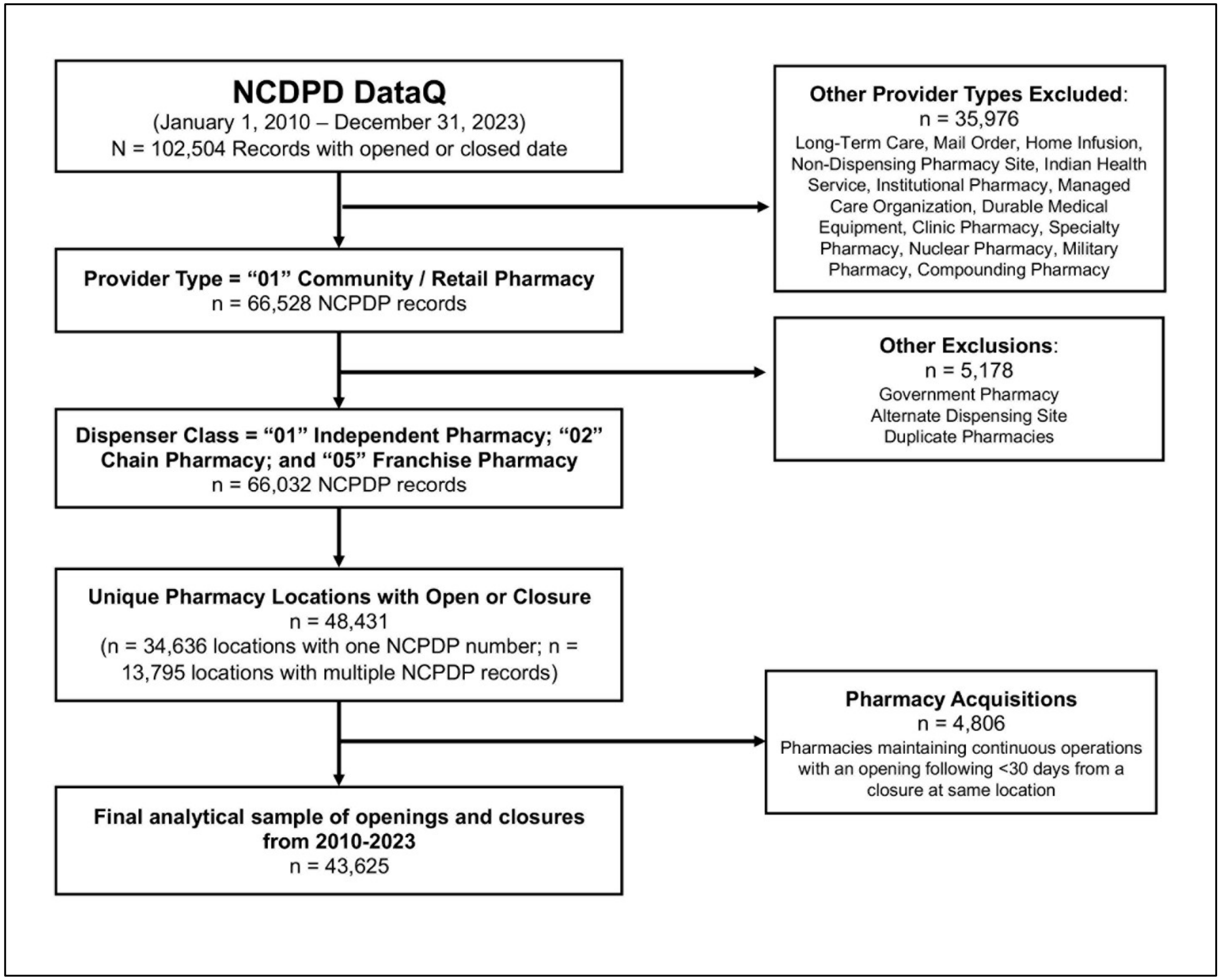

© 2025 Mattingly TJ et al. *JAMA Health Forum*.
