# Supplementary material for: Community Pharmacy Turnover and Context of Openings and Closings by Ownership Type
Source: JAMA Health Forum. 2025 Aug 1;6(8):e251988. doi: 10.1001/jamahealthforum.2025.1988 (PMC12317352; doi:10.1001/jamahealthforum.2025.1988)
Supplement: Supplement 2. — Data Sharing Statement [file jamahealthforum-e251988-s002.pdf]

Data Sharing Statement

Mattingly, II. Community Pharmacy Turnover and Context of Openings and Closings by Ownership Type. *JAMA Health Forum*. Published August 01, 2025.
doi:10.1001/jamahealthforum.2025.1988

Data
Data available: No

Additional Information
Explanation for why data not available: The pharmacy-level data used in this study was acquired from National Council for Prescription Drug Programs (NCPDP) and under a data use agreement with that entity.
